# Dietary pattern trajectories in French adults of the NutriNet-Santé cohort over time (2014–2022): role of socio-economic factors

**DOI:** 10.1017/S0007114524002514

**Published:** 2024-11-14

**Authors:** Hafsa Toujgani, Justine Berlivet, Florine Berthy, Benjamin Allès, Joséphine Brunin, Hélène Fouillet, Mathilde Touvier, Denis Lairon, François Mariotti, Julia Baudry, Emmanuelle Kesse-Guyot, the TRANSFood Consortium

**Affiliations:** 1 Université Sorbonne Paris Nord and Université Paris Cité, Inserm, INRAE, CNAM, Center of Research in Epidemiology and Statistics (CRESS), Nutritional Epidemiology Research Team (EREN), Bobigny 93017, France; 2 ADEME, Agence de l’Environnement et de la Maîtrise de l’Energie), Angers 49004, France; 3 Paris-Saclay University, UMR PNCA, AgroParisTech, INRAE, Paris 75005, France; 4 Aix Marseille Université, Inserm, INRAE, C2VN, Marseille 13005, France

**Keywords:** Longitudinal dietary changes, Gender-specific approach, Socio-economic factors, Diet quality

## Abstract

Despite the urgent need for plant-based dietary shifts, few studies have examined current diet trajectories using longitudinal data. This study analyzed dietary transitions of French adults over 8 years (2014-2022), assessing diet quality and the role of various socio-economic factors. Consumption data from 17 187 NutriNet-Santé cohort participants, weighted for the French Census, were collected via FFQ in 2014, 2018 and 2022. Adopting a gender-specific approach, consumption changes in twenty-three food groups were assessed over time. Diet quality was evaluated using the Comprehensive Diet Quality Index score, categorising foods into ‘healthy’ and ‘unhealthy’. Socio-economic analysis targeted four food groups (red meat (including fresh beef, pork, offal and lamb), processed meat (e.g. sausages, ham and bacon), legumes and whole-grain products), strongly linked to mortality risk and recognised as significant markers of the sustainable diet transition. All analyses were conducted using multi-adjusted mixed-effects models. Consumption of some healthy plant-based foods (nuts +59 %, legumes +22 %, whole-grain products +7 %) significantly increased over time, while consumption of some unhealthy foods (red meat −19 %, refined cereals −18 %, sweetened drinks −15 %) decreased. Conversely, consumption of prepared and mixed dishes (+16 %) and processed meat (+35 %) increased. These changes differed in magnitude between genders and translated into an improved diet quality score (Comprehensive Diet Quality Index). Occupational status was linked to longitudinal changes in food consumption, showing increased consumption of plant-based foods among students and higher socio-professional categories. Our findings provide accurate data on trends and factors for targeted initiatives, guiding strategic interventions for a sustainable dietary transition.

## Highlights

- Consumption of some healthy plant-based foods (legumes +22 %, nuts +59 %, whole-grain products +7 %) significantly increased over time (2014–2022), while consumption of some unhealthy foods (red meat −19 %, refined cereals −18 %, sweet drinks −15 %) decreased.
- Consumption of prepared and mixed dishes (+16 %) and processed meat (+35 %) increased during the study period.
- Diet quality (Comprehensive Diet Quality Index score) increased over time.
- Differences were observed between women and men in baseline consumption and in the magnitude of changes over time.
- Occupational status was linked to longitudinal changes in food consumption.
- Increased consumption of plant-based foods was observed among students and higher socio-professional categories.

The rate of climate change is accelerating at an alarming pace, outstripping current mitigation efforts and intensifying the need for swift and effective measures. In 2023, Europe experienced one of its hottest years on record^(1)^, underscoring the troubling reality that some planetary boundaries have been irreversibly crossed^(2–4)^. Additionally, food systems, which account for nearly one-third of global greenhouse gas emissions^(5)^, are often overlooked in climate policies^(6)^. Although some nationally determined contributions mention this sector, they focus on production, neglecting key factors like food waste and consumption habits, which are significant contributors to emissions^(6)^. Therefore, adopting a comprehensive approach to food systems to improve climate strategies is crucial, addressing all stages from production to sustainable consumption. In line with this, the 28th Conference of the Parties to the United Nations Framework Convention on Climate Change (COP28) Declaration on Food and Agriculture emphasises the need for stronger collaboration between key ministries (agriculture, climate, energy, environment, finance and health)^(7)^ to meet the Sustainable Development Goals by 2030^(8)^. Moreover, policymakers should consider local consumption patterns and cultural contexts to ensure that climate measures are both realistic and effective. In this context, consumer habits are crucial for effective transition strategies, as dietary shifts are increasingly necessary to mitigate climate change^(9,10)^, as noted in the latest Intergovernmental Panel on Climate Change report^(11)^. Reducing animal-based food consumption, which generates twice the emissions of plant-based alternatives^(12)^, is critical in lowering environmental impacts. For example, replacing red meat with poultry^(13)^ or increasing the proportion of plant-based proteins in the diet^(14)^ lessens the contribution to global warming.

A shift towards more plant-based diets is already observed in WHO member countries^(15)^, despite the relatively limited adoption of vegetarian and vegan diets^(16)^. Between 2009 and 2019, notable dietary changes were documented, particularly in France, where red meat consumption declined in favour of poultry and processed meats, predominantly among individuals aged 65 years and older. In contrast, those over 50 demonstrated an increased consumption of fruits and vegetables^(17)^. Nevertheless, these dietary shifts remain polarised, with some populations adopting more sustainable and health-promoting diets, while others continue to follow less favourable eating patterns. This polarisation can largely be attributed to socio-economic factors, as described in the Social Determinants of Health Framework, which emphasises the distinction between various levels of causality^(18)^, and the Nutrition Health Disparities Framework^(19)^, a recent adaptation of the Social Determinants of Health specifically focused on nutrition. For example, income levels are crucial in determining access to high-quality foods, contributing to nutritional inequalities^(20)^. According to Bennett’s law^(21)^, higher income facilitates a more diverse diet that includes animal proteins, while lower income is associated with increased carbohydrate consumption and a reduction in protein intake. Moreover, foods of lower nutritional value tend to be more affordable per calorie, making them more accessible to lower socio-economic groups^(20)^. Favourable dietary practices, such as higher consumption of fruits and vegetables, are often associated with a higher socio-economic status, particularly in relation to educational attainment^(22)^.

Despite advancements in the study of dietary behaviours, a substantial gap remains in understanding the relationships between these individual behaviours and socio-economic status. This deficiency is often due to the complex interplay of various internal and external factors influencing food choices^(19)^. In this context, our study aims to improve this understanding by characterising the changes in individual dietary consumption in France over the last decade while assessing diet quality. Furthermore, we will explore the associations between these dietary changes in the four sustainability-relevant food groups and individuals’ socio-economic status to better elucidate the connections between these two dimensions. Thus, the main objective of this analysis is to provide accurate data on the ongoing trends and the factors associated with these changes, intending to develop targeted initiatives tailored to specific food groups and subgroups within the population. This approach seeks to inform strategic and customised interventions that promote a sustainable dietary transition among the population.

## Methods and data

### Study population

The present study used longitudinal observational data from 2014 to 2022, using a sub-sample of the NutriNet-Santé study. Initiated in May 2009, the NutriNet-Santé study is an online-based cohort aimed at examining the factors influencing diets, nutritional status, physical activity and their relationships to health outcomes^(23)^. The study involves adult participants residing in France who have internet access, recruited on a voluntary basis. Participants are required to complete annual or biannual questionnaires covering socio-economic status, lifestyle, anthropometry, dietary and physical activity habits^(24)^. Additional questionnaires are periodically administered. Gender, occupational status, income, place of residence, physical activity levels and smoking habits are all self-reported using validated questionnaires^(24)^.

The NutriNet-Santé study complies with the principles outlined in the Helsinki Declaration and has received validation from both the Inserm Ethical Evaluation Committee (CEEI) (no. 0000388FWA00005831) and the National Committee for Information Technology and Freedom (CNIL) (nos. 908450 and 909216). The study is also registered on ClinicalTrials.gov (NCT03335644).

### Sociodemographic data

The self-reported individual characteristics, including gender, age, educational level (primary, secondary, post-secondary), employment status (unemployed, managerial staff, employee or manual labourer, self-employed or farmer, intermediate profession, student, retired) and monthly household income per consumption unit considering the household size and the age of its members (< 1200; 1200–1800; 1800–2700; > 2700 in euros per consumption unit per month)^(25)^, were collected at the baseline year 2014.

### Assessment of food group consumption

Food consumption data were collected in 2014, 2018 and 2022, through the use of an Organic FFQ encompassing a total of 264 organic and conventional food items, as described elsewhere^(26)^. For the present study, a classification into twenty-three food groups has been established based on their nutritional value and contents, as follows: red meat, poultry, processed meat, fish, eggs, dairy products (excluding milk), milk, animal substitutes, vegetables, fruits, fruit juice, legumes, whole-grain products, nuts, potatoes, refined cereals, prepared and mixed dishes (including sandwich, prepared foods such as pizza, hamburger, ravioli, panini, salted pancake), salty and sweetened fatty foods (including croissants, pastries, chocolate, biscuits, milky dessert, ice cream, honey and marmalade, cakes, chips, salted oilseeds, salted biscuits), sweetened drinks, hot drinks, alcohol, butter and plant-based fat. Total daily energy intake was calculated using the food composition table designed for the NutriNet-Santé study^(27)^.

### Diet quality data

Three dietary indexes were calculated for assessing the trend towards a healthy plant-based diet. The cDQI (Comprehensive Diet Quality Index) is designed to assess overall diet quality by evaluating plant and animal components^(28)^.

The pDQI (Plant-based Diet Quality Index) emphasises the quality of plant-based foods, dividing them into two categories: healthy foods (including whole-grain products, fruits, vegetables excluding potatoes, nuts, seeds and legumes, vegetable oils, coffee and tea) and foods to be consumed with moderation (such as refined grains, fruit juices, potatoes, sweetened beverages and sugary foods).

Similarly, the aDQI (Animal-based Diet Quality Index) evaluates the role of dietary quality of animal-origin foods. This index comprises two groups of items: healthy foods (such as fish, seafood, dairy products and poultry) and items to be restricted (including red meat, processed meat and eggs). Each food item is assigned a score ranging from 0 to 5 based on its alignment with the reference consumption. Consequently, the ultimate pDQI scores range from 0 to 55, and the aDQI scores range from 0 to 30. The comprehensive cDQI score is derived by summing the pDQI and aDQI scores, ranging from 0 to 85.

### Statistical analysis

This study included 17 187 participants who provided Organic FFQ data on food consumption during at least two collection periods (2014, 2018 and/or 2022). From an initial eligible population of 29 195 individuals who completed the FFQ in 2014, we selected those with a follow-up in either 2018 or 2022 to ensure longitudinal tracking. This approach accommodates missing data in mixed-effects models, allowing for the modelling of consumption trajectories with at least two follow-up points. Individuals who completed the FFQ in 2018 and/or 2022 but did not participate in 2014 were excluded from the analysis. The sample selection process is depicted in online Supplementary Fig. 1.

These participants had no missing information regarding sociodemographic factors (except for the non-mandatory monthly income question) and were not living overseas to permit the computation of a weighting procedure described below. Individuals classified as either underreporting or overreporting their energy intake, as detailed in a previous publication^(26)^, were excluded.

In order to correct the low generalisability of the findings from the sample of volunteers in the NutriNet-Santé cohort, the sample was corrected by weighting using the iterative proportional fitting procedure according to 2009 national census reports^(29)^ on age, occupational category, educational level, area of residence, presence of children (< 18 years) and marital status.

This method aims to enhance the representativeness of our sample within the French population. Consequently, a weighting factor was computed for each individual, reflecting the probability of their inclusion in a representative sample^(29)^.

Study population was described using mean (sd) or *n* % for continuous and categorical variables, respectively. The Pearson’s Chi-square test was used for categorical variables, while the ANOVA test was used for continuous variables.

To estimate changes in consumption of twenty-three food groups over time, mixed-effects models for repeated measurements were employed, adjusted for gender, age and total energy intake. Further details are outlined in online Supplementary Method 1.

We secondly aimed to examine socio-economic factors associated with the consumption of four food groups, namely, red meat, processed meats, legumes and whole-grain products, along with their longitudinal change. These food groups are strongly associated with mortality risk, both positively and negatively, based on data from the Global Burden of Disease^(30)^, and recognised as significant markers of the transition towards sustainable diets^(31–34)^. To achieve this, mixed-effects models similar to those used earlier were used, incorporating the relevant covariates, as described in online Supplementary Method 1.

This analysis was conducted on a sample of 16 239 individuals, for whom income data were available.

Furthermore, we computed the percentage variations relative to each food group, examining changes over time, across genders and socio-economic factors using the least squares means of adjusted consumption.

To assess the overall change in the quality of dietary patterns over time, we computed the cDQI indicator^(28)^, along with its two components, the aDQI and the pDQI reflecting the DQI of animal and plant-based foods, respectively. This approach allows for a meaningful classification of foods into ‘healthy’ and ‘unhealthy’ categories. Similar mixed-effects models as those employed earlier were utilised and detailed in online Supplementary Method 1.

## Results

### Sample characteristics

After sample weighting, the gender ratio was balanced between men (48 %) and women (52 %), with an average age of 48 (sd = 16) years old (Table 1). A detailed comparison of the differences between respondents and non-respondents is provided in online Supplementary Table 1.

**Table 1.** Participant characteristics, *n* 17 187, NutriNet-Santé Study^*,†^

	Whole sample		Women		Men		*P*
Unweighted *n*	*n* 17 187		*n* 12 479 (72·61 %)		*n* 4708 (27·39 %)		
Weighted *n*	*n* 17 187		*n* 8999 (52·36 %)		*n* 8188 (47·64 %)		
	Mean	sd	Mean	sd	Mean	sd	
Age	48·38	15·97	48·81	13·68	47·90	20·83	<0·0001
Education level	%		%		%		<0·0001
Primary	59·92		58·59		61·39		
Secondary	15·19		15·88		14·43		
Post-secondary	24·89		25·53		24·19		
Occupational position							<0·0001
Self-employed/farmer	4·45		2·42		6·67		
Managerial staff/intellectual profession	9·10		6·74		11·69		
Unemployed	8·85		12·63		4·69		
Employee, manual worker	31·19		30·53		31·90		
Students	4·49		4·60		4·40		
Intermediate professions	14·50		14·47		14·53		
Retired	27·42		28·61		26·12		
Monthly income per household unit							<0·0001
NA	6·58		8·49		4·48		
<1200€/C.U.	24·42		27·64		20·89		
1200–1800€/C.U.	30·91		28·53		33·52		
1800–2700€/C.U.	23·39		22·11		24·80		
>2700€/C.U.	14·70		13·23		16·31		
Place of residence							<0·0001
Rural community	23·99		25·62		22·19		
Urban unit (<20 000 inhabitants)	18·63		16·50		20·95		
Urban unit (20 000–200 000 inhabitants)	16·80		18·30		15·17		
Urban unit (>200 000 inhabitants)	40·58		39·57		41·70		
Smoking habits							<0·0001
Never smoker	50·16		55·60		44·17		
Former smoker	39·02		33·71		44·85		
Current smoker	10·83		10·69		10·98		
Physical activity							<0·0001
Low	20·30		20·26		20·35		
Moderate	31·16		31·68		30·59		
High	32·89		33·53		32·18		
Missing data	15·65		14·53		16·88		
BMI							<0·0001
Underweight (<18·5 kg/m^2^)	3·70		5·94		1·24		
Healthy (18·5–24·9 kg/m^2^)	55·75		58·55		52·67		
Overweight (25–29·9 kg/m^2^)	26·90		21·30		33·06		
Obese (≥30 kg/m^2^)	13·64		14·21		13·03		
	Mean	sd	Mean	sd	Mean	sd	
BMI	24·94	4·99	24·54	4·44	25·38	6·17	<0·0001

Abbreviation: C.U., consumption unit.

* Values are mean (sd) or % as appropriate; all data are weighted.

† *P* values calculated using ANOVA or *χ*^2^ test.

### Overall dietary change over 8 years

**Figure 1.**
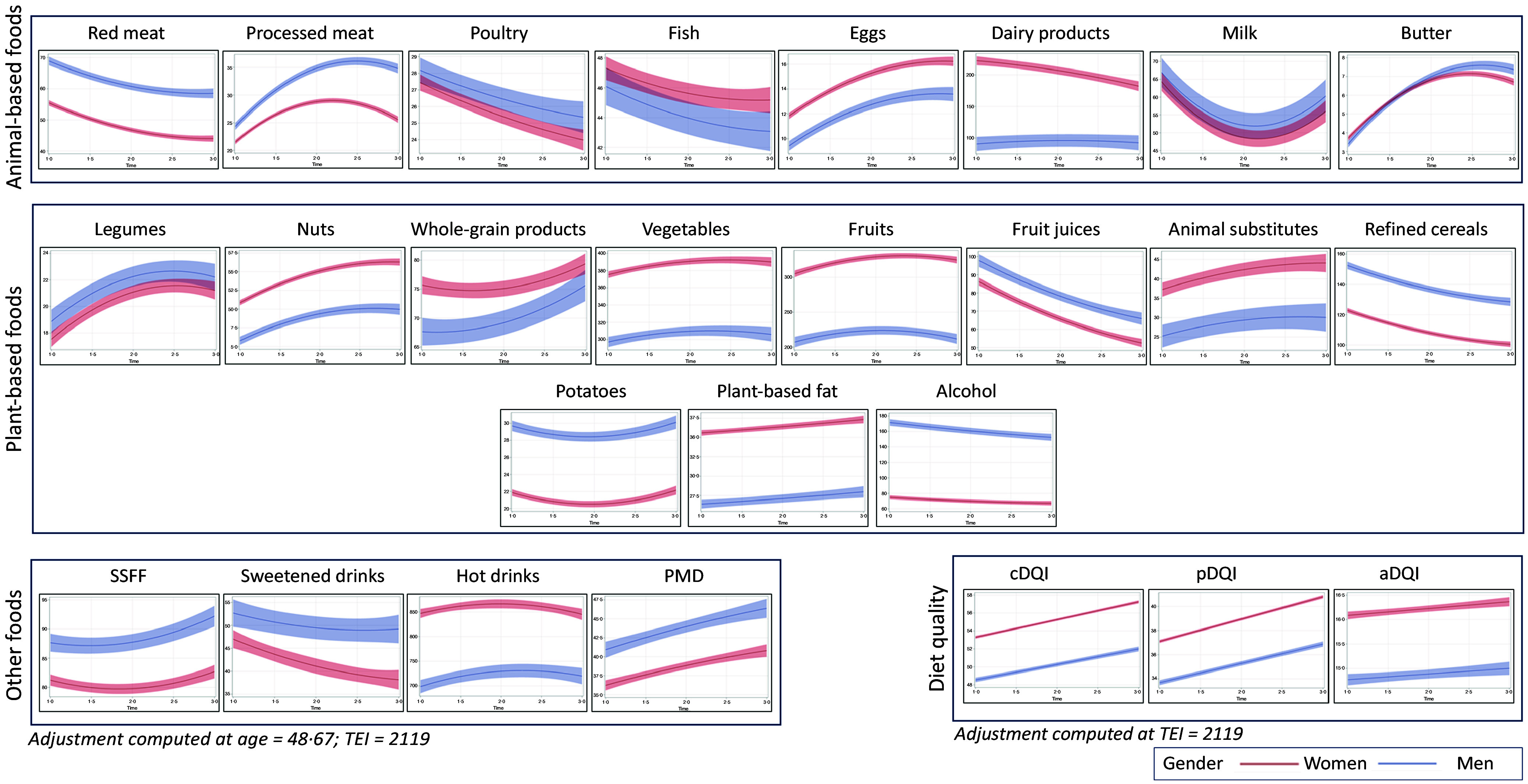
Evolution trajectories for food groups’ consumption and diet quality (2014–2022), *n* 17 187, NutriNet-Santé Study^1,2,3^. Food groups are formed as follows: red meat include beef, pork, offal and lamb; dairy products include yogurts, fresh cheese and cheese; animal substitutes include tofu, soy-based meat substitute and vegetable patties, soy-based yogurt and soy-based milk; vegetables include all vegetables and soups; fruit include fresh fruit, fruit in syrup and compote and dried fruit and seeds; fish include fatty and lean fish, molluscs and crustaceans; potatoes include other tubers; refined cereals include breakfast cereal low in sugar, bread, semolina and rice and pasta; SSFF (sweetened and salty fatty foods) include croissants, pastries, chocolate, biscuits, milky dessert, ice cream, honey and marmalade, cakes, chips, salted oilseeds and salted biscuits; PMD include sandwich, prepared foods such as pizza, hamburger, ravioli, panini, salted pancake, etc.; plant-based fat include plant-based oils and ready-to-use salad dressing, mayonnaise or cream-based sauces and sour cream and all fat-based sauces; hot drinks include tea, infusions and milk consumed with tea/coffee; sweetened drinks include fruit nectar, syrup, soda (with or without sugar) and plant-based beverages.^1^Abbreviations: TEI, total energy intake over time; PMD, prepared and mixed dishes; cDQI, Comprehensive Diet Quality Index; pDQI, plant-based Diet Quality Index; aDQI, Animal-based Diet Quality Index. ^2^For the twenty-three food groups, adjustments have been computed at age = 48·67 years and TEI = 2119 kcal/d. For the Diet Quality Indexes, adjustments have been computed at TEI = 2119 kcal/d. ^3^The x-axis represents time (1 = 2014; 2 = 2018; 3 = 2022), while the y-axis represents consumption in grams per d.

Significant changes in consumption patterns were observed over the past 8 years among the whole population for the majority of the twenty-three food groups examined (Fig. 1 and online Supplementary Table 2). The significant declines (*P* < 0·01) (online Supplementary Table 3) were in consumption of fruit juices (–40 %), red meat (–19 %), refined cereals (–18 %), sweetened drinks (–15 %), poultry (–12 %), milk (–12 %), alcohol (–12 %) and fish (–6 %). Conversely, food groups with the most noticeable increases (*P* < 0·01) included butter (+100 %), nuts (+59 %), eggs (+39 %), processed meat (+35 %), animal substitutes (+22 %), legumes (+22%), prepared and mixed dishes (+16 %) and whole-grain products (+7 %).

Consumption of vegetables (+4 %), fruits (+4 %) and salty and sweetened fatty foods (+3 %) slightly increased over time (*P* < 0·01), while consumption levels of fruits (+2 %), hot drinks (+2 %) and potatoes (+1 %) remained relatively stable (*P* < 0·01). However, the variations observed for dairy products (*P* ≈ 0·85) and plant-based fats (*P* ≈ 0·36) over time were not statistically significant.

Consistently with the increase in consumption of healthy foods, a growing cDQI score over time was observed (*P* < 0·01). The same was true for animal (aDQI) and plant (pDQI) components of the diet. It should be noted that these scores were higher for women compared with men (Fig. 1**)**.

### Gender-specific dietary change

Baseline consumption levels (2014) varied depending on gender (Fig. 1 and online Supplementary Tables 2 and 3). Women had higher consumption levels of healthy food groups than men (*P* < 0·01), including nuts (+85 % compared with men), fruits (+40 %), plant-based fats (+34 %), vegetables (+25 %), whole-grain products (+10 %). On the other hand, men had higher consumption levels of red meat (+23 % compared with women; *P* < 0·01), energy-dense foods (potatoes (+35 %; *P* < 0·01) and refined cereals (+27 %; *P* < 0·01)), prepared and mixed dishes (+15 %; *P* < 0·01), salty and sweet fatty products (+7 %; *P* < 0·01)) and alcohol (+119 %; *P* < 0·01).

Moreover, differences in food groups’ consumption over time were observed between genders (Fig. 1 and online Supplementary Tables 2 and 3). A widening discrepancy in the consumption of processed meat was noted, particularly with a notable increase among men (the gap went from +7 % in 2014 to +30 % in 2022, with a higher consumption among men; *P* < 0·01). Additionally, a more pronounced decline in sweetened drink consumption was observed among women (the gap went from +16 % in 2014 to +38 % in 2022, with a higher consumption among men; *P* ≈ 0·01).

### Socio-economic factors linked to the consumption of food groups highly associated with mortality risk

**Figure 2.**
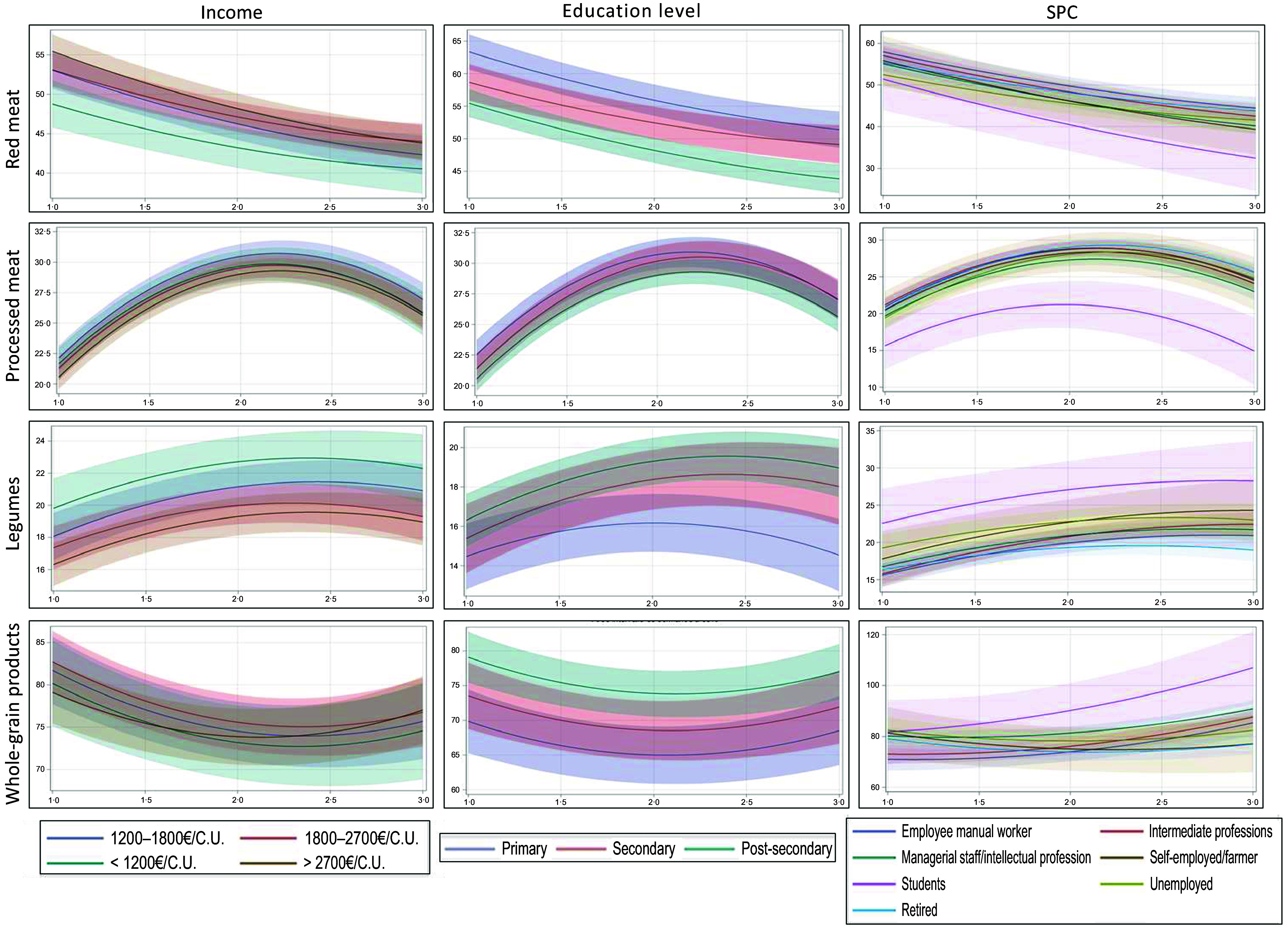
Socio-economic determinants of the consumption evolution for the four food groups strongly associated with mortality risk (red meat, processed meat, legumes and whole-grain products) between 2014 and 2022, *n* 17 187, NutriNet-Santé Study^1,2^. Abbreviation: SPC, Socio-Professional Category. ^1^ Adjustment computed at age = 48·8; TEI = 2133; gender = women; occupational status = retired; income >2700 €/CU; education = post-secondary. ^2^ The x-axis represents time (1 = 2014; 2 = 2018; 3 = 2022), while the y-axis represents consumption in grams per d.

Socio-economic factors, namely, income, education and occupational status, were associated with the consumption of the food groups examined in this study to varying degrees (Fig. 2 and online Supplementary Tables 4 and 5). All results were estimated with respect to the reference categories, including ‘highest income (> €2700 consumption unit per month)’ for income, ‘retired’ for occupational status and ‘post-secondary’ for educational level.

Income and education level were primary determinants of baseline consumption patterns, while occupational status played a significant role in influencing changes over time. Participants with higher education levels and lower incomes tended to consume more plant-based foods at the outset, and this trend intensified over time, particularly among students and individuals in higher socio-professional categories. Specifically, legume consumption at baseline was 22 % higher among individuals with the lowest incomes (*P* < 0·01), while whole-grain products consumption was 6 % higher among those with moderate incomes (*P* ≈ 0·02). Conversely, individuals with the lowest education levels exhibited the lowest baseline consumption of whole-grain products, with a 13 % reduction in consumption (*P* < 0·01).

Over the period from 2014 to 2022, the increase in legume consumption was notably more pronounced among participants in intermediate professions, showing a rise of 36 % (*P* < 0·01). For whole-grain products, the greatest increases were observed among students (+29 %; *P* < 0·01), those in intermediate professions (+23 %; *P* < 0·01), individuals in intellectual or managerial roles (+14 %; *P* < 0·01) and employees or manual workers (+20 %; *P* < 0·01).

Conversely, participants with lower education levels consumed more animal-based foods. More specifically, at baseline, red meat consumption was 14 % higher (*P* < 0·01) among individuals with the lowest education levels, while processed meat consumption was also highest in this group, with an 8 % greater consumption (*P* < 0·01). In contrast, individuals with the lowest incomes consumed 11 % less red meat (*P* < 0·01), whereas processed meat consumption was 6 % higher among those with moderate incomes (*P* < 0·01). Over time, the decline in red meat consumption from 2014 to 2022 was more pronounced among participants in intermediate professions (–20 %; *P* ≈ 0·03), intellectual or managerial professions (–21 %; *P* < 0·01) and high-income earners (–20 %). The gap in red meat consumption between the lowest and highest income groups narrowed over this period, decreasing from an 11 % difference in 2014 to 8 % in 2022 (*P* ≈ 0·04). For processed meat, the increase over time was more significant among those in intermediate professions (+32 %; *P* ≈ 0·01), intellectual or managerial professions (+34 %; *P* < 0·01) and students (+20 %; *P* ≈ 0·01).

## Discussion

### Towards healthier food consumption

Over the study period (2014–2022), we noted changes for most of the studied food groups’ consumption. A notable trend was the increased consumption of healthy plant-based foods (legumes, nuts and whole-grain products). Concurrently, there was a reduction in the consumption of unhealthy foods, encompassing both animal and plant sources (red meat, refined cereals, sweetened drinks and alcohol). A comparable trajectory was observed in the Netherlands, where a 20-year (1993–2015) cohort analysis revealed positive shifts in dietary practices within isoenergetic diets^(35)^. In contrast, findings from a Swedish study showed a deviation from Nordic dietary guidelines over time (2000–2016) among participants in the Northern Sweden Diet Database^(36)^. Furthermore, a similar trend towards more plant-based diets has been observed throughout the WHO European region^(15)^.

By extending the analysis to food groups, our research showed a decline in both red meat and poultry consumption. These findings could be interpreted in light of the French household meat purchase data for at-home consumption, revealing a declining trend of purchase volume of meat in both 2021 and 2022^(37)^. However, when considering the apparent consumption of meat, an upturn trend was observed over the same period, including out-of-home dining, as well as a significant 11·5 % surge in French meat imports^(37)^. Also, it has been shown that the decline in red meat consumption noticed in France from the mid-2000s (−12 % in 10 years) has been, to some extent, compensated by an increase in poultry consumption^(38)^, while our results indicate a decrease in all meat categories (except for processed meat). This could be partially attributed to certain specific characteristics within our population, despite efforts made to adjust for the French census. However, this might be seen in a different light, as a French survey (2010–2019) indicated that the substitution of protein sources has remained heterogeneous^(39)^. Thus, it is noteworthy that various approaches to quantifying food consumption produce notably distinct results, compromising the precision and relevance of comparisons. This disparity also applies to the composition of the considered food groups. Therefore, the use of individual consumption data provides a more precise and representative perspective of actual dietary habits.

Moreover, we observed an increase in the consumption of legumes, vegetables and fruits between 2014 and 2018, followed by a plateau in 2022. Indeed, the recent data from the Freshful Consumption Monitor^(40)^ revealed that twenty EU member states, including France in 2021, still consume quantities of fruit and vegetables below the WHO’s recommended daily consumption (at least 400 g/d). The hypothesis related to the context such as inflation and the Covid-19 pandemic may explain such a slowdown in the transition to a diet more in line with sustainable recommendations.

Through the use of a gender-specific approach with energy-adjusted consumption data, our results supported the existing literature^(41–43)^ according to which gender emerges as a significant determinant of dietary behaviours. We observed that men tended to consume a greater quantity of animal products, energy-dense foods and unhealthy items, while women exhibited a greater inclination towards the consumption of plant-based and healthy foods. Indeed, when investigating the obstacles to changing dietary behaviours, a notable gender-based contrast emerged in relation to portion sizes and preferences^(44)^. Previous works demonstrated that women are more inclined to adopt diets rich in plant-based foods^(45,46)^, while an American study indicated that men tended to report less healthy lifestyles compared with women, marked by a reduced willingness to cut back on meat consumption^(44)^. In fact, gender is among the most influential predictor of meat consumption levels^(47,48)^, and this gender-related effect could be explained, to some extent, by the perception associating meat consumption with masculinity^(44)^. Moreover, the consumption trends of certain unhealthy foods were advancing more rapidly over time among men than women. This is evident in the increasing consumption of processed meat and butter, as well as the slower decline in sweetened drinks and fruit juices among men. This pattern was also observed for legumes, where the increase was more pronounced among women.

Furthermore, the observed changes in dietary patterns in our study were reflected in an improvement in the quality of diets, as evidenced by the upward trajectories of the cDQI, aDQI and pDQI scores, with higher levels among women compared with men. The slight increase in the aDQI score could be attributed to a more pronounced decline in unhealthy animal-based foods compared with healthy ones. Regarding plant-based foods, the increased pDQI score might be explained by the increase in healthy plant-based sources like legumes, nuts and whole-grain products, contrasted with a simultaneous decrease in unhealthy plant-based items, such as refined cereals, fruit juices and alcohol. A comparable result was achieved in the previously mentioned Dutch cohort, with an improvement in the Dutch Healthy Diet Index 2015 score for both men (+11 %) and women (+13 %) over two decades, mainly explained by an increased consumption of (shell)fish and nuts/seeds/nut paste^(35)^. Yet, an Australian study involving adults over 55 years old (2010–2014) showed that the improvement in the quality of diets was evident only among men^(49)^.

Also, the observed trends align with recommendations for promoting a sustainable dietary transition, as the mentioned plant-based foods provide dual benefits for both health and the environment^(9,33,50–53)^. However, the Dutch study mentioned above noted no significant reduction in greenhouse gas emissions despite a shift towards more sustainable diets. This underscores the ongoing necessity for significant reductions in the consumption of animal-based foods, especially red meat^(54)^, to achieve diets that are both healthier and more environmentally friendly^(35)^.

### Dietary transition and socio-economic status

Our results highlighted that individuals with higher education levels tended to consume less red meat and more whole-grain products, while those with the lowest educational attainment demonstrated a higher consumption of processed meat. Indeed, education level has an impact on the experience of barriers to dietary change^(55)^. Lower education levels are well-established correlates of poorer dietary patterns, while higher education levels at baseline are predictive of a shift towards healthier dietary patterns^(48)^. Besides, a prior study on the same cohort NutriNet-Santé revealed that socially favoured individuals, with a higher level of education, exhibited healthier lifestyles and displayed a significantly elevated Sustainable Diet Index score^(56)^. Another study found that individuals with a higher level of education showed a preference for a vegetarian diet^(47)^. These patterns may be partially attributed to the association between education levels, nutritional knowledge and environmental awareness. However, uncertainty persists about the effectiveness of nutritional knowledge in public health nutrition due to the ongoing ambiguity surrounding the relationship between the nature of knowledge and dietary behaviours^(57)^. Yet, although of limited influence, nutritional knowledge can play an important role in the adoption of healthier dietary behaviours^(57)^.

Besides, our study revealed that income significantly influences dietary choices. Low-income individuals consumed less red meat when compared with those with a higher income. Yet, individuals with a higher income demonstrated a more substantial decline in meat consumption over time, narrowing the consumption gap compared with those with a lower income. These results align with a study comparing meat consumption trends based on income in developed countries and the Kuznets environmental curve^(58)^. The comparison indicated an inverted U-shaped relationship, suggesting that beyond a certain income threshold, increased awareness of environmental and health concerns leads to reduced meat consumption^(59)^.

Furthermore, our findings revealed that legumes were more consumed by individuals with the lowest incomes and students. While being affordable, this food group plays a significant role in sustainable dietary transition, with environmental and health co-benefits^(30,60–62)^. However, it is still under-consumed in high-income countries^(63)^, and various barriers have been pointed out in the literature. For instance, challenges related to sensory and preparation time and knowledge were observed in Denmark and the UK, whereas participants from Germany, Spain and Poland encountered hurdles linked to digestive problems when incorporating pulses^(64)^.

This prompts us to consider the significance of ‘perception’ in shaping dietary preferences. Indeed, adopting a sustainable diet is often perceived as costly, and studies revealed that affluent individuals are more inclined to embrace sustainable dietary choices. The above-mentioned study on the NutriNet-Santé cohort^(56)^ revealed that within the subset of individuals with the highest Sustainable Diet Index scores, 56·74 % reported high incomes (>2700€ per consumption unit). Conversely, affluent individuals tend to consume more meat than their counterparts with lower incomes. Additionally, traditionally linked to lower-income groups^(65)^, sustainable foods like legumes are more consumed by individuals with limited financial resources. In this context, two strategies for improving the perception of sustainable diets emerge: optimising the ‘price signal’ of sustainable foods by incorporating externalities into costs and implementing communication campaigns and practical workshops to promote awareness of the advantages of sustainable dietary choices.

### Strengths and limitations

As participants in the NutriNet-Santé cohort are voluntary, our sample is not representative of the French population. Nonetheless, we have sought to mitigate this issue by weighting the data. Despite a selection bias, our longitudinal study design offers unique insights into individual-level dietary changes over time, a perspective not achievable through repeated cross-sectional surveys, thus providing valuable contributions to understanding temporal trends in dietary patterns. Second, our study did not account for potential changes in occupational position and income over time, as we assumed that distinctions among the different classes remained consistent throughout the study period. However, our study is among the few depicting recent individual changes in dietary behaviours in a French cohort study. The substantial size of our sample enables us to explore a diverse range of profiles, including individuals who have already initiated the transition to a sustainable diet. This grants us the opportunity to analyse their profiles and gain insights from their trajectories. Moreover, analysing changes based on socio-economic determinants provides relevant information to develop targeted initiatives tailored to specific food groups and subgroups within the population.

### Conclusion

In conclusion, over the past 8 years (2014–2022), the dietary habits of French adults have witnessed an increase in the consumption of healthy, plant-based foods and a decline in the consumption of unhealthy foods of both animal and plant origin. The extent of these changes varied between genders and resulted in an enhanced diet quality score (cDQI). Income, education level and occupational position appear to be determining factors of these observed changes. Our results offer accurate information on current trends and associated factors that could be used to formulate targeted initiatives for specific food groups and subgroups within the population. This approach seeks to direct strategic and personalised interventions, promoting a sustainable transition in dietary habits.

## Supporting information

Toujgani et al. supplementary material Toujgani et al. supplementary material

## Supplementary material

For supplementary material/s referred to in this article, please visit https://doi.org/10.1017/S0007114524002514.

## Data Share Statement

Analytic code will be made available upon request pending. Researchers from public institutions can submit a collaboration request including information on the institution and a brief description of the project to collaboration@etude-nutrinet-sante.fr. All requests will be reviewed by the steering committee of the NutriNet-Santé study. If the collaboration is accepted, a data access agreement will be necessary and appropriate authorisations from the competent administrative authorities may be needed. In accordance with existing regulations, no personal data will be accessible.

## Registration of Clinical Trial

Trial registration number: NCT03335644

URL of registration: https://clinicaltrials.gov/study/NCT03335644?id=NCT03335644&rank=1
