# Supplementary material for: Dietary pattern trajectories in French adults of the NutriNet-Santé cohort over time (2014–2022): role of socio-economic factors
Source: Br J Nutr. 2024 Oct 17;132(9):1184–93. doi: 10.1017/S0007114524002514 (PMC11617108; doi:10.1017/S0007114524002514)
Supplement: Toujgani et al. supplementary material [file S0007114524002514sup001.docx]

# Online Supplemental material

**Supplemental Method 1: Mixed-effects models**

To estimate changes in consumption of 23 food groups over time, mixed-effects models for repeated measurements were employed. The general model for each food group was specified as:

$$Y_{ij}= \beta_{0}+\beta_{1}T_{ij}+\beta_{2}S_{i}+\beta_{3}A_{i}+\beta_{4}E_{ij}+\beta_{5}\left( T_{ij}\times S_{i} \right)+\beta_{6}T_{ij}^{2}+\mu_{i}+\varepsilon_{ij}$$

Where:

Y_ij_: Food group consumption for subject i at time point j.

T_ij_: Time variable (fixed effect) for subject i at time point j

S_i_: Sex (fixed effect) for subject i

A_i_: Age at baseline (fixed effect) for subject i

E_ij_: Total energy intake (repeated measure) for subject i at time point j

µ_i_: Random effects for subject i, including at least random intercept and slope

ε_i,j_​: Residual error term for subject i at time point j

Time was modelled to serve both as a fixed effect, capturing the average effect across the entire population, and as a random effect, accounting for individual variability in the temporal trajectory. An interaction term between time and gender was added to estimate the potential differences in evolution trajectories between men and women. The polynomial term Time² was introduced to capture potential non-linearity in the trajectory. The spatial power law SP(POW)(time) of the SAS MIXED procedure was utilized to model the correlation structure of repeated measures, due to variations in correlation over time, attributed to the inconsistent time intervals between successive questionnaires. For random effects, various structures for the variance-covariance matrix were explored, and the optimal one (unstructured) was chosen.

In the second part of our analysis, which focuses on examining socioeconomic factors associated with the consumption of four food groups, namely red meat, processed meats, legumes, and wholegrain products, we utilized mixed-effects models similar to those employed earlier, incorporating the relevant covariates as follows:

$$Y_{ij}= \beta_{0}+\beta_{1}T_{ij}+\beta_{2}S_{i}+\beta_{3}A_{i}+\beta_{4}E_{ij}+\beta_{5}Ed_{i}+\beta_{6}O_{i}+\beta_{7}I_{i}+\beta_{8}\left( T_{ij}\times S_{i} \right)+\beta_{9}\left( T_{ij}\times Ed_{i} \right)+\beta_{10}\left( T_{ij}\times I_{i} \right)+\beta_{11}\left( T_{ij}\times O_{i} \right)+\beta_{12}T_{ij}^{2}+\mu_{i}+\varepsilon_{ij}$$

Where additional terms include:

Ed_i_: Education level at baseline (fixed effect) for subject i

O_i_: Occupational status at baseline (fixed effect) for subject i

I_i_: Income at baseline (fixed effect) for subject i

Interaction terms between each of the covariates and time were incorporated into the model to capture their impact on consumption over time.

Similar mixed-effects models were utilized to model the trajectory of each of the the cDQI indicator, and its two components, the aDQI and the pDQI, as follows:

$$cDQI_{i,_{j}}=\beta_{0}+\beta_{1} T_{i},_{j}+\beta_{2} S_{i}+\beta_{3} E_{i},_{j}+\beta_{4} {(T}_{i},_{j}\times S_{i})+\mu_{i}+\varepsilon_{i},_{j}$$

**Supplemental Table 1: Sociodemographic Characteristics Across Study Waves^1,2,3^**

|  | **2014** | **2018**  **Resp.** | **2018**  **Non-resp.** | **P** | **2022**  **Resp.** | **2022**  **Non-resp.** | **P** | **2014_2018_2022**  **Resp.** | **2014_2018_2022**  **Non-resp.** | **P** |
| --- | --- | --- | --- | --- | --- | --- | --- | --- | --- | --- |
| Unweighted n | 17,187 | 15,548 | 1,639 |  | 10,734 | 6,453 |  | 9,095 | 8,092 |  |
| **Sex** |  |  |  | <0.0001 |  |  | 0,10 |  |  | 0,10 |
| Women | 52.36 | 53.07 | 46.8 |  | 51.81 | 53.1 |  | 53.05 | 51.76 |  |
| Men | 47.64 | 46.93 | 53.2 |  | 48.19 | 46.9 |  | 46.95 | 48.24 |  |
| **Age** | 48.38  (15.97) | 48.48  (16.02) | 47.59  (15.47) | <0.0001 | 49.44 (14.43) | 49.94 (18.13) | <0.0001 | 49.90  (14.19) | 47.07  (17.63) | <0.0001 |
| **Education level** |  |  |  | 0.0008 |  |  | <0.0001 |  |  | <0.0001 |
| Primary | 59.92 | 59.88 | 60.21 |  | 61.96 | 57.15 |  | 62.39 | 57.8 |  |
| Secondary | 15.19 | 15.52 | 12.63 |  | 12.38 | 19.01 |  | 12.32 | 17.65 |  |
| Post-secondary | 24.89 | 24.6 | 27.16 |  | 25.66 | 23.85 |  | 25.29 | 24.55 |  |
| **Occupational position** |  |  |  | <0.0001 |  |  | <0.0001 |  |  | <0.0001 |
| Self-employed/farmer | 4.45 | 4.6 | 3.22 |  | 3.91 | 5.17 |  | 4.08 | 4.76 |  |
| Managerial staff/intellectual profession | 9.10 | 8.69 | 12.26 |  | 10.74 | 6.87 |  | 10.36 | 8.02 |  |
| Unemployed | 8.85 | 9.34 | 5.04 |  | 11.46 | 5.29 |  | 13.05 | 5.24 |  |
| Employee. manual worker | 31.19 | 30.52 | 36.3 |  | 28.67 | 34.59 |  | 26.78 | 34.96 |  |
| Students | 4.49 | 4.34 | 5.74 |  | 2.67 | 6.98 |  | 1.91 | 6.72 |  |
| Intermediate professions | 14.50 | 13.94 | 18.83 |  | 15.1 | 13.68 |  | 14.18 | 14.77 |  |
| Retired | 27.42 | 28.56 | 18.6 |  | 27.44 | 27.41 |  | 29.63 | 25.54 |  |
| **Monthly income per household unit** |  |  |  | <0.0001 |  |  | <0.0001 |  |  | <0.0001 |
| NA | 6.58 | 6.83 | 4.65 |  | 6.44 | 6.77 |  | 6.88 | 6.32 |  |
| < 1200€/C.U. | 24.42 | 25.27 | 17.87 |  | 24.44 | 24.4 |  | 26.07 | 23.01 |  |
| 1200 - 1800€/ C.U. | 30.91 | 29.16 | 44.51 |  | 29.13 | 33.33 |  | 25.33 | 35.7 |  |
| 1800 - 2700€/ C.U. | 23.39 | 23.84 | 19.91 |  | 23.54 | 23.19 |  | 24.44 | 22.5 |  |
| >2700€/ C.U. | 14.70 | 14.91 | 13.07 |  | 16.46 | 12.31 |  | 17.29 | 12.47 |  |
| **Place of residence** |  |  |  | <0.0001 |  |  | <0.0001 |  |  | <0.0001 |
| Rural community | 23.99 | 24.61 | 19.16 |  | 24.39 | 23.43 |  | 25.69 | 22.52 |  |
| Urban unit (<20,000 inhabitants) | 18.63 | 19.29 | 13.4 |  | 18.62 | 18.64 |  | 19.91 | 17.53 |  |
| Urban unit (20,000 to 200,000 inhabitants) | 16.80 | 16.3 | 20.77 |  | 15.92 | 18 |  | 14.73 | 18.59 |  |
| Urban unit (>200,000 inhabitants) | 40.58 | 39.8 | 46.67 |  | 41.06 | 39.93 |  | 39.68 | 41.36 |  |

Abbreviations: C.U., consumption unit; Resp., respondents ; Non-resp., Non- respondents.

^1^Values are mean (SD) or % as appropriate, all data are weighted.

^2^P-values, calculated using ANOVA or Chi² test, indicate the significance of the differences between respondents and non-respondents for each wave of the study.

**Supplemental Table 2: Parameters of the mixed-effects models modeling the evolution trajectories of food groups (2014 to 2022), n = 17,187, NutriNet-Santé Study^1,2^**

^1^Abbreviations: TEI, Total energy intake over time; SSFF, Sweetened / Salty and Fatty Foods; PMD, Prepared and Mixed Dishes.

^2^Values are of the β-coefficients (95% CI) of the mixed-effects models of the evolution trajectories of food groups. Models have been adjusted for sex, age and TEI.

| **Food groups** | **Intercept** | **Age** | **TEI** | **Time** | **Sex (Men vs Women)** | **Time x Sex** | **Time x Time** |
| --- | --- | --- | --- | --- | --- | --- | --- |
| **Alcohol** | -38.22 (-48.25;-28.19) | 1.18 (1.03;1.33) | 0.03 (0.03;0.03) | -9.03 (-14.22;-3.84) | 102.44 (96.5;108.39) | -5.81 (-7.95;-3.67) | 1.3 (-0.01;2.6) |
| **Animal substitutes** | 10.23 (2.39;18.06) | -0.33 (-0.43;-0.23) | 0.02 (0.01;0.02) | 10.32 (4.86;15.77) | -10.95 (-15.45;-6.45) | -1.03 (-3.08;1.03) | -1.72 (-3.1;-0.35) |
| **Butter** | -9.14 (-9.68;-8.6) | 0.03 (0.02;0.04) | 0 (0;0) | 7.91 (7.49;8.32) | -0.79 (-1.1;-0.47) | 0.48 (0.32;0.65) | -1.6 (-1.71;-1.5) |
| **Dairy products** | 1.5 (-9.67;12.66) | 0.58 (0.43;0.73) | 0.09 (0.08;0.09) | -0.69 (-7.92;6.54) | -61.27 (-68.16;-54.38) | 8.44 (5.65;11.23) | -1.85 (-3.66;-0.03) |
| **Eggs** | -9.31 (-10.49;-8.13) | 0.1 (0.09;0.11) | 0 (0;0.01) | 7.16 (6.22;8.1) | -2.3 (-2.93;-1.67) | -0.11 (-0.44;0.22) | -1.24 (-1.48;-1) |
| **Fish** | -10.07 (-13.28;-6.87) | 0.46 (0.42;0.5) | 0.02 (0.02;0.02) | -3.52 (-5.76;-1.28) | -0.77 (-2.73;1.18) | -0.44 (-1.25;0.37) | 0.61 (0.05;1.18) |
| **Fruit juice** | 95.53 (87.45;103.61) | -0.51 (-0.6;-0.42) | 0.02 (0.02;0.02) | -31.54 (-37.39;-25.69) | 10.72 (4.33;17.1) | 0.89 (-1.94;3.71) | 3.68 (2.22;5.14) |
| **Fruits** | -221.42 (-241.22;-201.63) | 4.06 (3.84;4.28) | 0.13 (0.13;0.13) | 67.3 (51.45;83.15) | -90.26 (-102.2;-78.32) | -7.6 (-12.46;-2.75) | -14.37 (-18.34;-10.39) |
| **Hot drinks** | 432.4 (384.52;480.29) | 2.94 (2.5;3.39) | 0.1 (0.09;0.11) | 81.3 (36.39;126.21) | -159.98 (-178.98;-140.98) | 11.56 (2.91;20.21) | -20.54 (-31.82;-9.27) |
| **Legumes** | 3.09 (0.66;5.53) | -0.23 (-0.26;-0.2) | 0.01 (0.01;0.01) | 8.68 (6.76;10.59) | 1.5 (0.03;2.97) | -0.16 (-0.83;0.5) | -1.71 (-2.19;-1.23) |
| **Milk** | 54.05 (43.4;64.7) | -0.37 (-0.5;-0.24) | 0.03 (0.03;0.03) | -50.06 (-57.74;-42.39) | 1.76 (-4.71;8.23) | 0.84 (-2.02;3.71) | 11.48 (9.55;13.41) |
| **Nuts** | -11.54 (-12.99;-10.1) | 0.03 (0.01;0.05) | 0.01 (0.01;0.01) | 8.79 (7.81;9.76) | -4.45 (-5.31;-3.6) | -0.63 (-1.05;-0.2) | -1.52 (-1.76;-1.27) |
| **PMD** | 18.21 (15.71;20.71) | -0.45 (-0.48;-0.42) | 0.02 (0.02;0.02) | 3.59 (1.81;5.37) | 4.24 (2.21;6.28) | 0.44 (-0.55;1.43) | -0.33 (-0.77;0.11) |
| **Plant-based fat** | -3.76 (-5.39;-2.12) | 0.08 (0.06;0.1) | 0.02 (0.02;0.02) | 0.6 (-0.69;1.88) | -9.13 (-10.05;-8.22) | -0.07 (-0.52;0.37) | 0.07 (-0.26;0.39) |
| **Potatoes** | -0.97 (-2.67;0.73) | 0.07 (0.05;0.09) | 0.01 (0.01;0.01) | -5.85 (-7.13;-4.57) | 7.74 (6.72;8.77) | 0.08 (-0.42;0.57) | 1.5 (1.18;1.82) |
| **Poultry** | 18.62 (16.5;20.74) | -0.24 (-0.27;-0.22) | 0.01 (0.01;0.01) | -2.91 (-4.56;-1.26) | 0.41 (-0.88;1.71) | 0.32 (-0.31;0.95) | 0.3 (-0.12;0.71) |
| **Processed meat** | -15.15 (-16.97;-13.33) | -0.14 (-0.16;-0.12) | 0.01 (0.01;0.01) | 23.16 (21.76;24.55) | -0.36 (-1.34;0.61) | 3.19 (2.71;3.68) | -5.28 (-5.63;-4.93) |
| **Red meat** | 2.72 (-0.76;6.21) | 0.22 (0.17;0.26) | 0.03 (0.03;0.03) | -17.54 (-20;-15.09) | 12.94 (10.66;15.22) | 0.52 (-0.51;1.55) | 2.95 (2.34;3.57) |
| **Refined cereals** | 104.36 (98.07;110.64) | -1.27 (-1.35;-1.19) | 0.05 (0.05;0.05) | -25.81 (-30.2;-21.43) | 30.45 (26.47;34.43) | -0.83 (-2.58;0.92) | 3.66 (2.56;4.76) |
| **SSFF** | 17.9 (13.97;21.83) | -0.3 (-0.35;-0.25) | 0.04 (0.04;0.04) | -8 (-10.66;-5.33) | 4.87 (2.48;7.26) | 1.54 (0.48;2.6) | 2.18 (1.51;2.85) |
| **Sweet drinks** | 57 (48.22;65.79) | -0.85 (-0.94;-0.77) | 0.02 (0.02;0.02) | -10.29 (-17.92;-2.67) | 2.99 (-1.83;7.81) | 2.69 (0.61;4.76) | 1.47 (-0.45;3.38) |
| **Vegetables** | -74.64 (-91.21;-58.06) | 3.06 (2.87;3.25) | 0.13 (0.12;0.13) | 39.42 (26.57;52.27) | -75.06 (-85.65;-64.48) | -2.99 (-8.47;2.5) | -8.02 (-11.25;-4.79) |
| **Wholegrain products** | -4.49 (-10.95;1.96) | -0.04 (-0.11;0.04) | 0.04 (0.04;0.04) | -7.84 (-12.75;-2.94) | -10.16 (-13.94;-6.38) | 2.14 (0.48;3.8) | 2.42 (1.19;3.65) |

**Supplemental Table 3: Food group consumption over time (2014 to 2022), n = 17,187, NutriNet-Santé Study^1,2^**

| **Food groups** | **Whole sample** |  |  | **Men** |  |  | **Women** |  |  |
| --- | --- | --- | --- | --- | --- | --- | --- | --- | --- |
|  | **2014** | **2018** | **2022** | **2014** | **2018** | **2022** | **2014** | **2018** | **2022** |
| **Processed meat** | 21.59 (21.27;21.92) | 29.4 (28.99;29.81) | 29.1 (28.63;29.58) | 22.37 (21.81;22.93) | 33.18 (32.48;33.89) | 32.86 (32.05;33.66) | 20.82 (20.47;21.16) | 25.62 (25.18;26.05) | 25.35 (24.84;25.87) |
| **Legumes** | 16.47 (15.97;16.97) | 18.96 (18.47;19.46) | 20.03 (19.53;20.53) | 17.19 (16.33;18.05) | 19.29 (18.43;20.15) | 20.47 (19.62;21.33) | 15.75 (15.22;16.27) | 18.64 (18.12;19.16) | 19.59 (19.05;20.13) |
| **Plant-based fat** | 30.67 (30.38;30.97) | 30.9 (30.54;31.25) | 32.28 (31.88;32.68) | 26.21 (25.70;26.72) | 26.03 (25.42;26.64) | 27.75 (27.07;28.42) | 35.14 (34.82;35.45) | 35.76 (35.39;36.14) | 36.82 (36.39;37.25) |
| **SSFF** | 78.98 (78.13;79.83) | 80.17 (79.17;81.18) | 81.33 (80.55;82.11) | 81.61 (80.15;83.07) | 84.56 (82.84;86.28) | 85.22 (83.90;86.55) | 76.36 (75.44;77.27) | 75.79 (74.71;76.86) | 77.43 (76.56;78.30) |
| **Fish** | 47.88 (47.19;48.57) | 47.82 (47.18;48.46) | 45.22 (44.39;46.04) | 47.17 (45.98;48.36) | 47.27 (46.16;48.38) | 44.13 (42.73;45.53) | 48.59 (47.86;49.32) | 48.37 (47.70;49.04) | 46.3  (45.41;47.19) |
| **Potatoes** | 24.98 (24.64;25.31) | 24.66 (24.29;25.02) | 25.18 (24.74;25.62) | 28.71 (28.13;29.29) | 28.83 (28.21;29.45) | 29.08 (28.34;29.83) | 21.25 (20.90;21.60) | 20.48 (20.10;20.86) | 21.28 (20.80;21.75) |
| **Poultry** | 25.56 (25.15;25.97) | 23.24 (22.80;23.68) | 22.52 (21.94;23.10) | 25.86 (25.15;26.57) | 23.8 (23.04;24.56) | 23.15 (22.18;24.13) | 25.26 (24.83;25.69) | 22.69 (22.23;23.15) | 21.89 (21.27;22.52) |
| **PMD** | 33.32 (32.57;34.06) | 37.03 (36.59;37.48) | 38.5 (37.99;39.01) | 35.62 (34.38;36.86) | 40.01 (39.30;40.72) | 40.93 (40.12;41.74) | 31.01 (30.19;31.84) | 34.06 (33.54;34.58) | 36.07 (35.44;36.70) |
| **Refined cereals** | 125.85 (124.18;127.52) | 110.54 (109.40;111.69) | 102.77 (101.05;104.48) | 140.7 (137.83;143.57) | 125.51 (123.53;127.49) | 117.25 (114.34;120.17) | 110.99 (109.23;112.75) | 95.58 (94.385;96.778) | 88.28 (86.448;90.129) |
| **Alcohol** | 128.16 (126.08;130.24) | 122.1 (119.92;124.28) | 112.9 (110.64;115.17) | 176.03 (172.44;179.62) | 167.08 (163.33;170.84) | 153.95 (150.08;157.82) | 80.29 (78.097;82.485) | 77.12 (74.839;79.405) | 71.86 (69.417;74.313) |
| **Butter** | 3.47 (3.37;3.56) | 7.15 (7.01;7.29) | 6.923 (6.76;7.07) | 3.25 (3.09;3.41) | 7.47 (7.23;7.70) | 7.12 (6.86;7.38) | 3.68 (3.58;3.78) | 6.84  (6.69;6.98) | 6.72  (6.55;6.89) |
| **Sweet drinks** | 43.01 (41.35;44.67) | 38.23 (36.30;40.17) | 36.63 (35.04;38.22) | 46.23 (43.37;49.09) | 43.29 (39.97;46.61) | 42.49 (39.77;45.21) | 39.79 (38.04;41.54) | 33.18 (31.15;35.20) | 30.77 (29.05;32.49) |
| **Wholegrain products** | 68.01 (66.77;69.26) | 70.38 (69.02;71.75) | 72.64 (71.12;74.16) | 64.72 (62.56;66.87) | 67.17 (64.81;69.52) | 71.09 (68.51;73.68) | 71.31 (69.99;72.63) | 73.6 (72.17;75.04) | 74.18 (72.54;75.82) |
| **Fruit juice** | 85.76 (83.86;87.66) | 68.07 (66.35;69.79) | 51.66 (50.10;53.22) | 91.61 (88.35;94.88) | 74.06 (71.11;77.01) | 58.16 (55.50;60.82) | 79.9 (77.90;81.91) | 62.08 (60.28;63.88) | 45.16 (43.47;46.84) |
| **Milk** | 58.86 (56.62;61.09) | 48.03 (45.67;50.40) | 51.58 (47.40;55.77) | 60.33 (56.47;64.18) | 50 (45.93;54.08) | 53.57 (46.47;60.67) | 57.38 (55.02;59.75) | 46.06 (43.58;48.55) | 49.59 (45.11;54.07) |
| **Nuts** | 8.4 (8.122;8.685) | 11.49 (11.14;11.84) | 13.38 (12.94;13.81) | 5.898 (5.412;6.384) | 8.32 (7.715;8.929) | 10.22 (9.489;10.95) | 10.9 (10.61;11.20) | 14.66 (14.29;15.03) | 16.53 (16.06;17.00) |
| **Eggs** | 11.18 (10.99;11.37) | 14.23 (13.94;14.52) | 15.52 (15.20;15.85) | 10.13 (9.807;10.45) | 12.93 (12.44;13.43) | 14.44 (13.89;14.98) | 12.23 (12.03;12.43) | 15.53 (15.22;15.83) | 16.61 (16.26;16.96) |
| **Dairy products** | 182.58 (180.45;184.72) | 177.39 (174.70;180.07) | 175.85 (173.38;178.32) | 156.66 (152.97;160.35) | 155.84 (151.23;160.45) | 157.64 (153.44;161.85) | 208.51 (206.25;210.76) | 198.93 (196.12;201.74) | 194.06 (191.39;196.73) |
| **Red meat** | 61.36 (60.6;62.12) | 54.09 (53.33;54.86) | 49.87 (49.11;50.63) | 67.63 (66.33;68.93) | 61.54 (60.24;62.85) | 56.47 (55.17;57.77) | 55.09 (54.28;55.89) | 46.65 (45.84;47.45) | 43.27 (42.44;44.10) |
| **Animal substitutes** | 26.11 (24.39;27.82) | 32.21 (30.31;34.11) | 31.78 (29.41;34.15) | 22.22 (19.39;25.06) | 25.23 (22.08;28.39) | 26.28 (22.37;30.19) | 29.99 (28.02;31.96) | 39.19 (37.05;41.34) | 37.28 (34.57;39.99) |
| **Vegetables** | 351.79 (348.26;355.33) | 358.51 (354.59;362.42) | 365.73 (361.16;370.30) | 312.92 (306.89;318.94) | 315.04 (308.39;321.69) | 324.38 (316.73;332.04) | 390.66 (386.80;394.53) | 401.98 (397.76;406.19) | 407.07 (401.96;412.19) |
| **Hot drinks** | 767.17 (758.20;776.14) | 796.5 (787.69;805.30) | 781.7 (770.72;792.68) | 711.84 (696.90;726.78) | 736.32 (721.68;750.96) | 736.9 (718.70;755.11) | 822.49 (812.32;832.67) | 856.67 (846.72;866.62) | 826.5 (814.00;839.00) |
| **Fruits** | 276.40 (272.32;280.49) | 293.65 (289.32;297.98) | 288.37 (284.34;292.41) | 229.9 (222.87;236.94) | 238.11 (230.68;245.54) | 235.33 (228.46;242.21) | 322.9 (318.59;327.21) | 349.18 (344.65;353.71) | 341.42 (337.05;345.78) |

^1^Abbreviations: SSFF, Sweetened / Salty and Fatty Foods; PMD, Prepared and Mixed Dishes.

^2^Values are the least squares means (CI) of adjusted consumptions (for sex, age and total energy intake).

**Supplemental Table 4: Parameters of mixed-effects models modeling the socioeconomic determinants of the consumption over time (2014 to 2022), for the 4 food groups strongly associated with mortality risk (red meat, processed meat, legumes, and whole products), n =17 187, NutriNet-Santé Study^1,2^**

|  | **Red meat** | | **Processed meat** | | **Legumes** | | **Wholegrain products** | |
| --- | --- | --- | --- | --- | --- | --- | --- | --- |
|  | **Estimate** | **Pvalue** | **Estimate** | **Pvalue** | **Estimate** | **Pvalue** | **Estimate** | **Pvalue** |
| **Intercept** | 4.17 (-1.78;10.12) | 0.16 | -16.58 (-19.47;-13.68) | < 0.0001 | -1.09 (-4.95;2.77) | 0.58 | -1.12 (-11.51;9.26) | 0.83 |
| **Time** | -11.61 (-14.25;-8.97) | < 0.0001 | 26.35 (24.81;27.9) | < 0.0001 | 8 (5.88;10.12) | < 0.0001 | -17.8 (-22.93;-12.67) | < 0.0001 |
| **Age** | 0.1 (0.03;0.17) | 0.01 | -0.19 (-0.22;-0.15) | < 0.0001 | -0.14 (-0.18;-0.09) | < 0.0001 | 0.13 (0.01;0.25) | 0.04 |
| **Total Energy Intake** | 0.03 (0.03;0.03) | < 0.0001 | 0.01 (0.01;0.01) | < 0.0001 | 0.01 (0.01;0.01) | < 0.0001 | 0.04 (0.04;0.04) | < 0.0001 |
| **Sex (Men vs Women)** | 13.29 (10.97;15.62) | < 0.0001 | -0.09 (-1.11;0.92) | 0.86 | 0.99 (-0.56;2.55) | 0.21 | -12.36 (-16.43;-8.29) | < 0.0001 |
| **Time x Sex (Men vs Women)** | 0.34 (-0.62;1.31) | 0.48 | 3.19 (2.69;3.7) | < 0.0001 | 0.23 (-0.47;0.93) | 0.52 | 3.02 (1.23;4.81) | < 0.0001 |
| **Income** |  |  |  |  |  |  |  |  |
| < 1200€/C.U. | -8.39 (-12.29;-4.48) | < 0.0001 | 1.57 (-0.14;3.28) | 0.07 | 3.58 (0.97;6.2) | 0.01 | 2.84 (-3.99;9.68) | 0.41 |
| 1200 - 1800€/C.U. | -2.82 (-5.69;0.06) | 0.05 | 1.72 (0.45;2.98) | 0.01 | 1.65 (-0.27;3.58) | 0.09 | 4.6 (-0.44;9.64) | 0.07 |
| 1800 - 2700€/C.U. | -3.57 (-6.17;-0.98) | 0.01 | 0.98 (-0.16;2.12) | 0.09 | 1.4 (-0.34;3.13) | 0.11 | 5.58 (1.04;10.12) | 0.02 |
| >2700€/C.U. | ref |  | ref |  | ref |  | ref |  |
| **Time x Income** |  |  |  |  |  |  |  |  |
| < 1200€/C.U. | 1.71 (0.07;3.35) | 0.04 | -0.47 (-1.33;0.39) | 0.29 | -0.08 (-1.28;1.11) | 0.89 | -1.77 (-4.81;1.27) | 0.25 |
| 1200 - 1800€/C.U. | 0.44 (-0.78;1.65) | 0.48 | -0.13 (-0.77;0.5) | 0.68 | 0.1 (-0.78;0.99) | 0.82 | -1.99 (-4.24;0.26) | 0.08 |
| 1800 - 2700€/C.U. | 1.26 (0.16;2.35) | 0.02 | -0.25 (-0.82;0.33) | 0.40 | -0.36 (-1.15;0.44) | 0.38 | -1.95 (-3.98;0.08) | 0.06 |
| >2700€/C.U. | ref |  | ref |  | ref |  | ref |  |
| **Education** |  |  |  |  |  |  |  |  |
| < Baccalaureate | 8.05 (5.28;10.82) | < 0.0001 | 2.3 (1.09;3.51) | 0.0001 | -0.55 (-2.4;1.3) | 0.56 | -9.62 (-14.47;-4.78) | < 0.0001 |
| Baccalaureate | 2.16 (-0.92;5.23) | 0.17 | 0.52 (-0.83;1.87) | 0.45 | -0.93 (-2.99;1.13) | 0.38 | -5.78 (-11.17;-0.39) | 0.03 |
| > Baccalaureate | ref |  | ref |  | ref |  | ref |  |
| **Time x Education** |  |  |  |  |  |  |  |  |
| < Baccalaureate | -0.16 (-1.31;0.99) | 0.78 | -0.31 (-0.91;0.29) | 0.31 | -1.29 (-2.13;-0.45) | 0.002 | 0.37 (-1.76;2.51) | 0.73 |
| Baccalaureate | 1.04 (-0.26;2.34) | 0.12 | 0.31 (-0.37;0.99) | 0.38 | 0 (-0.95;0.95) | 0.10 | 0.23 (-2.18;2.65) | 0.85 |
| > Baccalaureate | ref |  | ref |  | ref |  | ref |  |
| **Occupational status** |  |  |  |  |  |  |  |  |
| Employee/manual worker | 3.45 (-0.23;7.13) | 0.07 | 1.01 (-0.62;2.64) | 0.22 | -2.05 (-4.49;0.38) | 0.10 | -16.34 (-22.76;-9.92) | < 0.0001 |
| Intermediate professions | 3.12 (-0.32;6.55) | 0.07 | 1.5 (-0.02;3.02) | 0.05 | -2.55 (-4.82;-0.28) | 0.02 | -14.38 (-20.36;-8.39) | < 0.0001 |
| Managerial staff/intellectual profession | 1.37 (-1.84;4.59) | 0.40 | 0.02 (-1.41;1.45) | 0.97 | -0.73 (-2.84;1.39) | 0.50 | -5.22 (-10.81;0.37) | 0.07 |
| Unemployed | -3.29 (-7.61;1.03) | 0.13 | -1.31 (-3.23;0.6) | 0.18 | 2.37 (-0.51;5.25) | 0.11 | 2.32 (-5.23;9.87) | 0.55 |
| Self-employed/farmer | 2.86 (-5.56;11.28) | 0.50 | 0.65 (-3.01;4.3) | 0.73 | -0.43 (-6.06;5.19) | 0.88 | 3.38 (-11.35;18.1) | 0.65 |
| Students | -0.32 (-11.12;10.47) | 0.95 | -2.02 (-6.77;2.73) | 0.40 | 4.75 (-2.46;11.96) | 0.20 | -11.04 (-29.94;7.86) | 0.25 |
| Retired | ref |  | ref |  | ref |  | ref |  |
| **Time x Occupational status** |  |  |  |  |  |  |  |  |
| Employee. manual worker | -0.93 (-2.38;0.51) | 0.20 | -0.66 (-1.42;0.09) | 0.08 | 1.33 (0.28;2.39) | 0.01 | 8.21 (5.54;10.89) | < 0.0001 |
| Intermediate professions | -1.47 (-2.81;-0.13) | 0.03 | -0.88 (-1.58;-0.18) | 0.01 | 2.02 (1.04;2.99) | < 0.0001 | 8.32 (5.84;10.81) | < 0.0001 |
| Managerial staff/intellectual profession | -1.67 (-2.9;-0.43) | 0.01 | -0.89 (-1.54;-0.25) | 0.01 | 1.16 (0.27;2.06) | 0.01 | 6.34 (4.05;8.62) | < 0.0001 |
| Unemployed | 0.35 (-1.45;2.15) | 0.70 | 0.16 (-0.78;1.11) | 0.73 | 0.57 (-0.74;1.88) | 0.40 | 1.02 (-2.31;4.36) | 0.55 |
| Self-employed/farmer | -2.43 (-5.88;1.01) | 0.17 | -0.73 (-2.52;1.07) | 0.43 | 1.92 (-0.59;4.43) | 0.13 | -1.09 (-7.48;5.3) | 0.74 |
| Students | -3.68 (-8.08;0.72) | 0.10 | -2.9 (-5.21;-0.59) | 0.01 | 1.52 (-1.71;4.74) | 0.36 | 13.68 (5.49;21.87) | < 0.0001 |
| Retired | ref |  | ref |  | ref |  | ref |  |
| **Time x Time** | 1.45 (0.84;2.06) | < 0.0001 | -5.95 (-6.32;-5.59) | < 0.0001 | -1.67 (-2.16;-1.17) | < 0.0001 | 4.19 (3;5.38) | < 0.0001 |

^1^Values are of the β-coefficients (95% CI) of the mixed-effects models of the evolution trajectories of food groups. Models have been adjusted for sex, age, TEI and socioeconomic variables (Income, education and occupational status).

^2^P-values are those of β-coefficients.

**Supplemental Table 5: Food group consumption over time (2014 to 2022) in each socioeconomic category, n = 17,187, NutriNet-Santé Study^1^**

|  | **Red meat** | |  |  | **Processed meat** | |  |  | **Legumes** | |  |  | **Wholegrain products** | |  |  |
| --- | --- | --- | --- | --- | --- | --- | --- | --- | --- | --- | --- | --- | --- | --- | --- | --- |
|  | **2014** | | **2018** | **2022** | **2014** | **2018** | | **2022** | **2014** | **2018** | | **2022** | **2014** | **2018** | | **2022** |
| **Income** |  | |  |  |  |  | |  |  |  | |  |  |  | |  |
| < 1200€/C.U. | 57.00 (54.35;59.65) | | 50.85 (48.41;53.29) | 47.07 (44.52;49.62) | 21.85 (20.81;22.88) | 29.10 (27.80;30.41) | | 28.81 (27.24;30.38) | 19.43 (17.87;21.00) | 22.46 (20.94;23.98) | | 23.95 (22.18;25.72) | 64.93 (60.93;68.93) | 71.16 (66.94;75.39) | | 69.59 (64.58;74.60) |
| 1200 - 1800€/C.U. | 61.74 (59.56;63.91) | | 53.45 (51.45;55.46) | 49.08 (46.98;51.18) | 22.22 (21.37;23.06) | 30.14 (29.07;31.21) | | 29.04 (27.75;30.33) | 17.59 (16.30;18.87) | 20.98 (19.73;22.22) | | 22.35 (20.89;23.81) | 66.96 (63.67;70.25) | 70.98 (67.51;74.44) | | 72.66 (68.52;76.79) |
| 1800 - 2700€/C.U. | 61.88 (59.74;64.02) | | 54.39 (52.43;56.36) | 50.32 (48.27;52.37) | 21.39 (20.56;22.23) | 29.02 (27.97;30.08) | | 28.25 (26.98;29.52) | 17.51 (16.25;18.77) | 19.41 (18.18;20.63) | | 22.31 (20.88;23.73) | 68.10 (64.87;71.34) | 71.67 (68.26;75.08) | | 73.49 (69.44;77.53) |
| >2700€/C.U. | 64.24 (62.12;66.36) | | 55.77 (53.83;57.71) | 51.42 (49.41;53.43) | 21.01 (20.18;21.83) | 28.35 (27.30;29.40) | | 28.41 (27.16;29.66) | 15.95 (14.70;17.20) | 19.11 (17.90;20.32) | | 20.94 (19.54;22.34) | 64.03 (60.83;67.23) | 69.77 (66.39;73.16) | | 73.77 (69.80;77.75) |
| **Education** |  | |  |  |  |  | |  |  |  | |  |  |  | |  |
| Primary | 65.65 (63.42;67.89) | | 57.03 (54.98;59.08) | 52.41 (50.26;54.55) | 22.31 (21.45;23.18) | 30.00 (28.90;31.10) | | 29.66 (28.34;30.99) | 16.76 (15.44;18.08) | 17.92 (16.64;19.19) | | 20.41 (18.91;21.91) | 60.63 (57.27;63.99) | 65.70 (62.16;69.25) | | 67.57 (63.35;71.80) |
| Secondary | 61.20 (58.80;63.60) | | 53.29 (51.09;55.49) | 49.90 (47.60;52.20) | 21.48 (20.55;22.41) | 28.83 (27.65;30.02) | | 29.12 (27.70;30.54) | 17.19 (15.77;18.61) | 20.29 (18.91;21.66) | | 22.33 (20.73;23.94) | 63.64 (60.02;67.26) | 69.58 (65.76;73.40) | | 69.90 (65.36;74.44) |
| Post-secondary | 57.66 (55.85;59.48) | | 49.25 (47.58;50.93) | 44.99 (43.24;46.74) | 20.58 (19.86;21.29) | 27.92 (27.03;28.82) | | 27.39 (26.32;28.45) | 17.85 (16.78;18.92) | 21.73 (20.69;22.77) | | 23.01 (21.81;24.22) | 69.55 (66.79;72.31) | 74.78 (71.87;77.68) | | 75.99 (72.57;79.41) |
| **Occupational status** | |  |  |  |  |  | |  |  |  | |  |  |  | |  |
| Employee/manual worker | 64.90 (62.61;67.18) | | 56.45 (54.34;58.56) | 52.70 (50.54;54.87) | 22.64 (21.75;23.54) | 29.95 (28.82;31.07) | | 30.40 (29.07;31.73) | 15.32 (13.97;16.67) | 17.55 (16.24;18.87) | | 20.39 (18.89;21.89) | 57.41 (53.95;60.86) | 66.33 (62.67;69.98) | | 68.86 (64.62;73.10) |
| Intermediate professions | 63.48 (61.22;65.75) | | 55.09 (53.00;57.18) | 50.60 (48.50;52.70) | 23.04 (22.16;23.92) | 29.83 (28.71;30.95) | | 30.36 (29.07;31.65) | 15.54 (14.20;16.87) | 18.74 (17.43;20.04) | | 21.13 (19.68;22.58) | 58.87 (55.45;62.29) | 69.31 (65.67;72.94) | | 72.48 (68.37;76.59) |
| Managerial staff/intellectual profession | 61.22 (59.09;63.35) | | 52.96 (51.00;54.93) | 48.34 (46.36;50.32) | 21.35 (20.52;22.19) | 28.47 (27.41;29.52) | | 28.67 (27.46;29.89) | 16.32 (15.07;17.58) | 19.19 (17.96;20.41) | | 20.42 (19.06;21.78) | 66.81 (63.56;70.06) | 73.40 (69.97;76.84) | | 76.24 (72.35;80.13) |
| Unemployed | 59.22 (56.46;61.98) | | 52.27 (49.74;54.79) | 49.54 (46.91;52.17) | 20.82 (19.75;21.89) | 29.31 (27.94;30.68) | | 29.25 (27.61;30.89) | 18.63 (17.00;20.26) | 21.32 (19.74;22.90) | | 22.02 (20.18;23.86) | 68.63 (64.42;72.84) | 69.83 (65.40;74.26) | | 70.02 (64.76;75.28) |
| Self-employed/farmer | 62.49 (56.41;68.56) | | 52.45 (46.87;58.03) | 48.94 (43.26;54.61) | 22.98 (20.65;25.32) | 30.56 (27.54;33.58) | | 31.25 (27.74;34.76) | 17.22 (13.64;20.80) | 19.63 (16.13;23.12) | | 22.35 (18.41;26.29) | 66.78 (57.68;75.88) | 66.27 (56.59;75.96) | | 60.05 (48.92;71.17) |
| Students | 57.29 (49.96;64.61) | | 47.49 (40.74;54.25) | 41.77 (34.31;49.24) | 17.30 (14.44;20.16) | 23.86 (20.26;27.47) | | 20.76 (16.15;25.37) | 21.47 (17.16;25.79) | 26.07 (21.87;30.27) | | 29.17 (23.93;34.42) | 67.36 (56.33;78.40) | 80.48 (68.83;92.12) | | 86.72 (71.93;101.5) |
| Retired | 61.94 (60.33;63.55) | | 55.63 (54.13;57.13) | 51.79 (50.25;53.33) | 22.06 (21.41;22.71) | 30.46 (29.68;31.24) | | 30.37 (29.47;31.27) | 16.36 (15.41;17.31) | 17.34 (16.42;18.26) | | 17.95 (16.92;18.98) | 66.38 (63.90;68.86) | 64.53 (61.96;67.10) | | 63.71 (60.78;66.64) |

^1^Values are the least squares means (CI) of adjusted consumptions (for sex, age, total energy intake, education, income and occupational status).

***Supplemental Figure 1: Selection of the study sample***

n=37,685 had completed the organic food questionnaire between June and December 2014

n=37,305 had no missing covariates

n=35,196 were not under-reporter or over-reporter

n=34,453 were not living overseas

n=29,210 had available data regarding the place of purchase

Final sample of 2014 n=29,210

Among the 29,210 participants: 5 withdrew their consent; 6,453 completed both 2014 and 2018 FFQs ; 1,639 completed both 2014 and 2022 FFQs ; 9,095 completed all three FFQs (2014, 2018, 2022).
